# What Characterizes Employees with Emotional Exhaustion and Employees with Work Overload?

**DOI:** 10.3390/ijerph23060794

**Published:** 2026-06-12

**Authors:** Celine-Chantal Elster-Kann, Beate Muschalla

**Affiliations:** Institute of Psychology, Department of Clinical Psychology, Psychotherapy and Diagnostics, Technische Universität Braunschweig, Humboldtstraße 33, 38106 Braunschweig, Germany; b.muschalla@tu-braunschweig.de

**Keywords:** emotional exhaustion, job demands, occupational health, psychosocial work factor, sick leave, work ability, work overload, workplace

## Abstract

**Highlights:**

**Public health relevance—How does this work relate to a public health issue?**

- Emotional exhaustion can be an indicator and risk factor for various mental and somatic disorders, affecting work capacity and thereby increasing sickness rates and productivity loss.
- Changes in the world of work (e.g., digitalization and a growing shortage of skilled workers) increase the relevance of examining individual and structural determinants of work-related stress.

**Public health significance—Why is this work of significance to public health?**

- These representative data examine differences between employees with work overload and emotional exhaustion, providing a population-based perspective beyond specific occupational groups.
- There are group-specific characteristics across differently burdened employee groups, with several psychosocial work factors being more frequently observed across the overload and exhaustion groups.

**Public health implications—What are the key implications or messages for practitioners, policy makers, and/or researchers in public health?**

- This study highlights the potential importance of distinguishing work overload and illness-related exhaustion as separate risk factors for work capacity, suggesting the need for distinct intervention strategies.
- The findings suggest the relevance of focusing on psychosocial work factors, particularly social support and participation in work structuring, to prevent stress, with certain groups (e.g., individuals not engaged in their preferred occupation) requiring special attention.

**Abstract:**

Emotional exhaustion has been discussed as a major contributor to work ability problems, with substantial economic, individual, and social consequences. Research largely focuses on specific professions and sometimes overlooks that exhaustion and work overload problems are partly distinct. This study uses a differential analysis to explore working conditions and individual characteristics in employees with emotional exhaustion or perceived work overload, aiming to identify potential common risk factors. A representative German cross-sectional sample of 2289 employees aged 15–67, working at least 10 h per week, was analyzed. Employees with and without treatment for exhaustion, and with and without perceived work overload, were compared using variance analysis. Overloaded employees reported more work demands, while exhausted employees appear to be more often female and not in their preferred occupation. Several psychosocial work factors (e.g., responsibility) were more consistently associated with the overload and exhaustion groups than many of the physical work conditions. Employee characteristics such as openness and internal locus of control appeared to be similarly distributed across groups. Overload without exhaustion can be distinguished from combined exhaustion and overload, suggesting that work overload may occur with or without exhaustion, in relation to individual psychosocial resources. Preventive interventions for work ability may benefit from addressing overload as a distinct risk factor, besides illness-related exhaustion.

## 1. Introduction

For about 20 years, one of the most common reasons for long-term sick leave and work disability has been emotional exhaustion and mental health issues [1]. Individuals experiencing high levels of emotional exhaustion may not use the healthcare system as much as they should, which can raise their risk of further health decline. This may represent a major risk factor for work disability. Emotional exhaustion is associated with longer durations of work incapacity [2,3]. Therefore, it has become increasingly important for employers to examine contextual and personal risk factors related to work incapacity [4].

On the side of personal risk factors, emotional exhaustion is a common core symptom of many mental health issues [5,6]. Emotional exhaustion refers to feelings of being overwhelmed and drained. It comes along with reduced well-being and impaired occupational functioning. This state of emotional exhaustion is associated with a perceived inability to mobilize one’s resources effectively. Persons with mental health issues, such as anxiety or mood disorders, regularly have problems at work due to exhaustion [2,7].

On the side of contextual factors, research has found general risk factors for work disability, specifically an imbalance between work demands and resources, or a person-job-misfit [8,9,10,11]. A misfit of work demands (e.g., work overload) and available resources (e.g., insufficient team cooperation) may increase stress-related symptoms and emotional exhaustion [12].

The terms “work overload” and “emotional exhaustion” are often used together in the existing literature because there appears to be significant overlap in their relationships with other factors. However, Moreno-Martínez and Sánchez-Martínez [13], for example, recommend taking a closer look at the origins of both constructs. While emotional exhaustion can be understood as a psychological response to intense work-related stressors, work overload, according to the Job Demands-Resources model (JD-R model), reflects an imbalance between external demands and an individual’s coping abilities.

In case of work overload, job demands outweigh available resources. If this condition is persistent, it can compromise an individual’s ability to work. Accordingly, work overload can be experienced by multiple employees within the same work team and tends to result in compensation through heightened effort, while emotional exhaustion is more strongly influenced by personal factors, such as lower resilience, and tends to result in loss of motivation [13,14].

The person-job fit framework can be used to explain why people may respond differently to the same job demands. If the person-job fit is poor, the demands (JD-R model) are also perceived as less manageable. This raises the question of what may be important individual factors that may contribute to work exhaustion in some employees, but not in others [15].

A substantial body of empirical research based on this framework has identified predictors of perceived stress, emotional exhaustion, and, ultimately, burnout. Different individual and work-related characteristics have been examined. At the individual level, for example, researchers have found that higher levels of extraversion and hardiness serve as protective factors against emotional exhaustion [16,17]. At the level of working conditions, perception of increased workload, low autonomy, role ambiguity, and role overload represent risk factors for emotional exhaustion. Furthermore, some studies suggest indirect associations between overload and emotional exhaustion. Thus, high work overload has been associated with prolonged working hours [3], which may increase the risk of developing exhaustion [8,14].

Despite the overlap in contributing factors, it remains unclear whether employees experiencing work overload differ systematically from those experiencing emotional exhaustion [15]. Taken together, based on these findings, it can be assumed that individual and work-related characteristics interact in complex ways in influencing emotional exhaustion or work overload. Understanding overloaded or exhausted employees’ characteristics is crucial, as both overload and exhaustion may have significant effects on job satisfaction, work ability, organizational commitment, and turnover intentions [3,16,18,19].

Given current changes in the working world, increased flexibility and adjustment behavior are to be expected. According to the central JD-R and person-job fit models, not every employee experiencing high job demands will necessarily develop emotional exhaustion. Individual characteristics can act as a buffer against work overload and protect employees from health consequences such as emotional exhaustion. Therefore, an important question is, what distinguishes employees who experience work overload from those who also develop emotional exhaustion? Identifying typical characteristics of employees at risk of sick leave, particularly those experiencing emotional exhaustion or overload, may offer ways for preventive interventions [2,5,15,18,20].

### Research Gap and Research Questions

Although substantial research has been conducted on exhaustion, there remain limitations in the existing literature: Many studies are limited by their focus on specific professions (e.g., teachers, instructors, physicians, and nursing staff) or specific regional or organizational cultures. These points limit the generalizability of study findings [2,15]. Although some factors influencing emotional exhaustion have been identified, numerous other factors may still exist [17,21]. Work characteristics are often addressed only generally or indirectly in occupational psychology research [22,23,24]. So far, there have mainly been universal approaches, which also neglect that “exhaustion” and prominent work problems such as “overload” are partly different and may occur in different combinations.

The present representative study seeks to address this gap by comparing employees with illness-like exhaustion, employees who feel work overloaded, employees who are work overloaded and exhausted, and employees with neither problem. This comparative analysis will be done with a representative employee survey conducted by the Federal Institute for Vocational Education and Training (BIBB, Bonn, Germany) and the Federal Institute for Occupational Safety and Health (BAuA, Dortmund, Germany). The present analysis examines work and employee characteristics that may be associated with employees’ experience of being overburdened at work or exhausted. This gives rise to the following exploratory research question:

Which work-related and individual characteristics can be found in employees with emotional exhaustion and employees with work overload?

Therefore, the following four groups shall be compared: the “emotional exhaustion with perceived work-related overload”, “emotional exhaustion without overload”, “no emotional exhaustion but work-related overload”, and “neither exhaustion nor overload”.

A distinction is made between emotional exhaustion and overload to provide additional insights into why a high workload is associated with different psychological reactions. Based on the JD-R and person-job fit models, individuals differ in their attitudes, needs, and perceived person-job fit, which may influence whether job demands are perceived as work overload. At the same time, research on resilience suggests that employees do not react equally to stressful demands, meaning that not all individuals experiencing work overload necessarily develop psychological strain reactions, such as emotional exhaustion [10,13,14,15]. For this reason, the present study compares groups defined by combinations of emotional exhaustion and perceived work overload in terms of individual and work-related characteristics, without specifying directed hypotheses. Knowledge about characteristic patterns can help identify individuals at increased risk of work overload or emotional exhaustion, thereby enabling more targeted support.

## 2. Materials and Methods

### 2.1. Study Sample and Recruitment

This paper presents an analysis of the existing dataset from the BIBB/BAuA-Employment Survey (2017/18). In the first data collection in Germany, 20,012 employed persons were interviewed about characteristics of their work setting and employment. Because the main interview covered a very wide range of topics, a supplementary interview date was conducted that focused specifically on personality traits, resulting in 8010 employees who were additionally interviewed about their personality characteristics.

The dataset is representative of employed persons in Germany and is based on telephone interviews. The sample was randomly selected using a dual-frame approach that included both landline and mobile phone numbers. The Swedish key (Kish method) was applied for randomization. This method involves a systematic listing of all household members, from which the target person is randomly selected using a random number procedure.

Inclusion criteria for participation were engagement in paid employment for at least 10 h per week and a minimum age of 15 years. Vocational trainees were excluded from the outset. Further details on the sampling and data collection methodology can be found in the publicly available methodological report by the survey institute Kantar Public [25], which carried out the data collection on behalf of BIBB and BAuA [25,26].

### 2.2. Grouping Variables

For the present analysis, two grouping variables were chosen. First, the variable “Have you been in treatment for emotional exhaustion in the past 12 months” was selected, which participants answered with either “yes” or “no”. We used this variable because, in accordance with “evidence-based medicine” and therapy guidelines, treatment is prescribed and implemented only if there is a clinically relevant diagnosis. Consequently, it can be presumed that this question excludes everyday feelings of “exhaustion.”

As a second variable, “experience of overload” was chosen. The exact wording of the question for this variable was: “And do you usually feel up to the demands of the amount of work or the workload, rather overchallenged or rather underchallenged?” Participants were given the following response options: “generally able to meet the demands”, “somewhat overwhelmed”, and “somewhat underchallenged”. For our analyses, we dichotomized the grouping variable into “no perceived overload” and “perceived overload”. The group labeled “no perceived overload” includes those who reported being “generally able to meet the demands” or “somewhat underchallenged”, since both are degrees of the same category: neither group feels overwhelmed. Using “somewhat underchallenged” would introduce yet another phenomenon into the analysis—one that is not the focus of the present paper: “boredom”.

As the dataset did not include a direct measure of current work ability—defined as an individual’s ability to meet job demands—perceived overload was used as an early indicator for work ability problems. Overload is closely related to work ability and is a known risk factor for work incapacity. The feeling of being overloaded with work is an indication that the demands exceed individual capacity, which can lead to high energy costs, reduced performance, and adverse health consequences if the situation persists. That means the demands exceed an employee’s individual capabilities at several ability levels (physical, mental, technical), resulting in a lower overall ability to work. The capacities become fully exhausted [10,23,27]. These two grouping variables—exhaustion and work overload—were utilized to carry out comparative analyses.

### 2.3. Sociodemographic and Work-Related Characteristics

Sociodemographic and work-related characteristics included sex and age, individuals’ occupational development and the current work context, qualification (highest level of general school education, vocational training), occupational sector and status, working hours, employment duration, and interruptions. Most of these characteristics are nominal, although some were assessed in terms of years or hours. Occupational sectors were classified according to the standard classification system “KldB 2010” [28].

#### 2.3.1. Working Conditions and Demands

Various work activities and demands were assessed, including problem-solving, multitasking necessities, physiological and psychological working conditions, and strain.

#### 2.3.2. Personality Characteristics and Performance

Personality characteristics were assessed with validated standard self-rating scales:

- Locus of control examines whether an individual perceives events as resulting from their own actions or as externally controlled. The validated scale IE-4, Internal-External-Control Perception Scale, was used. McDonalds Omega ω = 0.53 to 0.71, i.e., internal consistency ranging from insufficient to acceptable [29].
- Self-efficacy was assessed using the six-item self-efficacy scale. Cronbach’s α = 0.77, indicating acceptable to good internal consistency [30].
- Work addiction behavior, characterized by an irresistible inner drive to work very hard, was evaluated with the Dutch-Work-Addiction-Scale (DUWAS). DUWAS can be split into excessive and compulsive working. Both subscales typically achieve Cronbach’s α values ranging from 0.63 to 0.78, indicating acceptable to good internal consistency [31].
- The Big Five personality traits were shortly assessed with the BFI-S, “Big-Five-Inventory Short version”. Cronbach’s α values, ranging from 0.50 to 0.67, indicate marginally acceptable to good internal consistency [32].
- Procrastination, defined as the tendency to put things off, was captured via a single-item scale; reliability coefficients (for example, Cronbach’s α) are not applicable [33].
- Finally, the willingness to take risks was measured with the short scale R-1, “Short Scale for Readiness to Take a Risk”, *r*_tt_ = 0.74, indicating acceptable temporal stability [34].

All scales use a five-point response format or, if needed, were transformed (e.g., DUWAS) to a uniform five-point format to avoid switching between different response scales.

Additionally, a vocabulary test was done as a basic indicator of performance. The participants had one minute to name as many different animals as possible in this task. Cronbach’s α is at a value of 0.57 for the vocabulary test, indicating marginally acceptable internal consistency [35].

Commonly used scales were used. The reliability coefficients of each scale are acceptable, although not particularly high. This is a common challenge when measuring psychological constructs. It should be noted that scale length was taken into account, which in turn reduces the reliability coefficients [36].

Overall, there is evidence supporting internal validity.

### 2.4. Statistical Analysis

Data have been analyzed according to the research question, comparing the four above-defined groups. Descriptive statistics and group comparisons were computed with IBM SPSS Statistics (Version 31.0, IBM Corp., Armonk, NY, USA). The four groups (“emotional exhaustion with perceived work-related overload”, “emotional exhaustion without overload”, “no emotional exhaustion but work-related overload”, and “neither of these”) were compared using analysis of variance (ANOVA) with Bonferroni or Games-Howell post hoc tests. For all nominal and categorical variables, χ^2^-tests with post hoc pairwise comparisons based on case selection for each pair of groups were carried out. Effect sizes were estimated using partial eta squared (η^2^) or Cramérs’ V.

Before data analysis, cases were excluded because key variables needed to address the research questions of this paper were missing or responses were incomplete. About 70% of the participants had to be excluded because they were not asked about the grouping item “treatment for emotional exhaustion.” Another percentage of less than 1% did not provide any information regarding the grouping variable of overload or had an “invalid occupational code”, which is an important variable for the investigation of work-related issues. This results in a sample size of 2.289 employees with full data for the present analysis. This resulted in a final sample size of *n* = 2289.

## 3. Results

### 3.1. Sociodemographic Characteristics

The overall mean age was 47.7 years (SD = 10.7 years). Employees with emotional exhaustion were slightly older. Most of the participants are married (54.2%). The majority had about two children under the age of 18 living in the same household (Mean = 1.7, SD = 0.8). A total of 60.5% of the investigated have a university entrance qualification (A-Levels, Abitur).

The group of exhausted employees without perceived overload included 298 participants, while the group with exhaustion and overload comprised 268 participants. The largest group consisted of 1017 employees without emotional exhaustion and without perceived overload. Additionally, there were 706 participants in the group without exhaustion but with work overload (Table 1).

The overall correlation (Spearman correlation) between exhaustion and overload was r = −0.061, *p* < 0.01. This very low correlation suggests that exhaustion and overload are only weakly related.

Women were more frequently represented in the exhausted groups (>60% women) compared to the non-exhausted (52–54% women). The group that experiences neither exhaustion nor overload has the highest proportion of married (56%) individuals and the lowest proportion of divorced or widowed people (14%). Employees without exhaustion more frequently reported having a university entrance qualification (62%) compared to those with exhaustion (52–57%). All characteristics (sex, marital status, and general school education) have a small effect size.

Overall, 13.7% of the sample has a recognized disability. In particular, the exhausted reported a significantly higher proportion having a recognized disability (24–30%) compared to those without exhaustion (9–10%), with the highest effect size.

### 3.2. Work Characteristics

The investigated sample consists of 95.5% with a vocational qualification, 59.1% are higher managers or professionals, 10% hold a secondary job, and 56.8% work in shifts.

Employees with exhaustion were less often employed in their preferred occupation (63–66%) compared to non-exhausted employees (73–77%, Table 1).

Those who do not experience emotional exhaustion but do experience overload often pursue careers in the “personal services” sector (45%) and are least likely to work in the “commercial and business-related services” sector (27%) compared to the other groups. The overloaded groups also have a higher occupational status (65%) than the others. The non-overloaded are more often working in routine and intermediate occupations (26–30%). Employees with the most weekly working hours (48–53%, not significant) and less work–life balance are more often overloaded (40–44%).

Employees with perceived overload (no matter if exhausted or non-exhausted) report the worst conditions in most work demands, whether physical or psychological: in particular, managing personal knowledge gaps, small influence on work arrangements (e.g., workload allocation and decisions to take a break), as well as poor lighting conditions and frequent production targets or time constraints (Table 2). However, the individual demands seem to contribute only marginally to the explanation of observed differences, with a few exceptions showing small effects, particularly high time and performance pressure, and high frequency of working at maximum capacity.

The group, which is both emotionally exhausted and overloaded, reports working at maximum capacity (57%), being confronted with emotionally demanding situations (48%), and having problems detaching from work (67%) more often than the other groups. Furthermore, this group reports receiving help at work from colleagues and supervisors least often (57%, 33%) as well as positive feedback (13%). Moreover, they report more frequently than the other three groups that they are not involved in important processes in a timely manner (33%), that they often experience a lack of information necessary to perform tasks properly (26%), and that they feel that they are doing important work less often (68%, Table 2).

In contrast, there is the group experiencing neither exhaustion nor overload. Among other things, this group feels more integrated into the work community (79%) and reports receiving more support from colleagues (77%) and supervisors (54%) at work. At the same time, this group reports the least amount of time and performance pressure (52%), receives detailed instructions the least frequently (18%), and experiences interruptions the least often (52%).

**Table 1:** Sociodemographic and work-related characteristics of employees with or without work overload, and with or without treatment due to emotional exhaustion. German representative working sample (*N* = 2289). One-way ANOVA with Bonferroni-corrected post hoc comparisons, Chi-square test.

		Employed with Emotional Exhaustion		Employed Without Emotional Exhaustion		ANOVA and χ^2^ *p* Overall, Bonferroni-Corrected Post Hoc Group Comparisons With Significant Differences		
Perceived Overload		Without Perceived Overload (1) *n* = 298	With Perceived Overload (2) *n* = 268	Without Perceived Overload (3) *n* = 1017	With Perceived Overload (4) *n* = 706		Partial η^2^ and Cramérs’ V	All (*N* = 2289)
Sociodemographic and Work-Related Characteristics								
Sex	female	67.4%	63.1%	52.1%	54.4%	<0.001 1 vs. 3, 4 2 vs. 3, 4	0.111	56.1%
Age		49.2 (9.6)	49.7 (9.7)	47.0 (11.2)	47.3 (10.5)	0.892		47.7 (10.7)
Marital status	married	50.3%	50.4%	55.7%	55.1%	0.024 < 0.05 3 vs. 1, 2	0.063	54.2%
	single	26.2%	31.0%	29.8%	26.3%			28.4%
	divorced	19.1%	14.2%	12.6%	15.3%			14.5%
	widowed	4.0%	4.1%	1.6%	3.0%			2.6%
	registered partnership	0.0%	0.0%	0.4%	0.1%			0.2%
Number of children under 18 years living in the same household		1.8 (0.9)	1.6 (0.8)	1.7 (0.7)	1.7 (0.8)	0.085		1.7 (0.8)
The highest general secondary school education, university entrance qualification (German Abitur)		52.7%	56.7%	62.9%	61.6%	0.040 < 0.05 1 vs. 3	0.089	60.5%
Vocational qualification-vocational training or higher education (yes)		94.0%	95.5%	95.3%	96.6%	0.287		95.5%
Occupational sectors (KldB 2010)	production-related	14.1%	13.4%	14.8%	13.9%	0.002 < 0.01 4 vs. 1, 3	0.067	14.3%
	personal service	35.2%	42.2%	36.5%	44.6%			39.5%
	commercial and business-related service	34.9%	34.7%	34.1%	27.1%			32.1%
	IT and scientific service	4.0%	4.5%	6.8%	7.1%			6.2%
	other economic service occupations	11.7%	5.2%	7.8%	7.4%			7.9%
Occupational status (ESeC 5 classes)	not classifiable	9.1%	11.6%	11.7%	11.2%	<0.001 1 vs. 2, 4 3 vs. 2, 4	0.080	11.2%
	higher managers & professionals	52.3%	65.3%	55.2%	65.2%			59.1%
	intermediate occupations	18.5%	12.3%	16.4%	10.3%			14.3%
	small employers and self-employed	1.3%	0.4%	1.4%	1.4%			1.3%
	lower sales and services	6.0%	2.6%	5.2%	5.1%			5.0%
	routine occupations	12.8%	7.8%	10.1%	6.8%			9.2%
Secondary jobs	yes	12.4%	6.7%	10.3%	9.6%	0.146		10.0%
Total actual weekly working hours, including secondary jobs		40.6 (15.3)	52.6 (19.0)	44.1 (13.1)	47.9 (12.9)	0.079		45.3 (14.2)
Overtime practices	overtime pay	10.9%	9.2%	8.0%	10.4%	0.359		9.3%
	compensatory time	54.0%	43.4%	47.2%	40.8%			45.2%
	both	18.2%	19.1%	20.5%	21.7%			20.5%
	no overtime compensation	16.1%	27.0%	22.9%	25.3%			23.5%
Shift work (yes)		44.4%	62.7%	56.7%	59.1%	0.083		56.8%
Are you employed in your preferred occupation? (yes)		66.1%	62.7%	77.5%	72.9%	<0.001 1 vs. 3, 4 2 vs. 3, 4	0.088	72.9%
Duration of current employment in years		14.2 (11.4)	17.2 (11.7)	13.4 (11.3)	14.7 (11.3)	0.115		14.3 (11.4)
Total lifetime unemployment (in years)		2.2 (2.7)	2.1 (2.2)	2.0 (2.0)	2.0 (1.7)	0.819		2.1 (2.1)
Recognized disability (yes)		29.9%	23.5%	9.1%	9.8%	<0.001 1 vs. 3, 4 2 vs. 3, 4	0.161	13.7%

Note. Means (SD) and percentage of participants reported. ESeC: higher managers and professionals (class 1 and 2: large employers, higher managers, higher supervisors/technicians), intermediate occupations (class 3 and 6: intermediate occupations, lower supervisors and technicians), small employers and self-employed (class 4 and 5: non-agriculture and agriculture), lower sales and service (class 7), routine occupations (class 8 and 9: lower technical, routine).

**Table 2:** Working conditions and demands in employees with or without work overload, and with or without treatment due to emotional exhaustion. German representative working sample (*N* = 2289). One-way ANOVA with Bonferroni-corrected post hoc comparisons, Chi-square test.

		Employed with Emotional Exhaustion		Employed Without Emotional Exhaustion		ANOVA and χ^2^ *p* Overall, Bonferroni-Corrected Post Hoc Group Comparisons With Significant Differences		
Perceived Overload		Without Perceived Overload (1) *n* = 298	With Perceived Overload (2) *n* = 268	Without Perceived Overload (3) *n* = 1017	With Perceived Overload (4) *n* = 706		Partial η^2^ and Cramérs’ V	All (*N* = 2289)
Working Conditions and Demands								
Work–life balance	often	62.1%	44.4%	64.1%	39.8%	<0.001 1 vs. 2, 4 3 vs. 2, 4	0.050	54.0%
	sometimes	21.8%	28.7%	22.0%	31.4%			25.7%
	rarely	11.4%	20.1%	11.7%	23.2%			16.2%
	never	3.7%	6.0%	1.4%	5.0%			3.3%
	no response	1.0%	0.7%	0.8%	0.6%			0.7%
Standby duty/on-call duty or work on call (yes)		23.5%	22.4%	26.3%	26.2%	0.478		25.4%
Occasional remote work (yes)		40.3%	43.0%	43.4%	47.8%	0.337		44.3%
**Occupational activities**								
Frequent problem-solving		78.9%	84.7%	81.0%	89.4%	<0.001 4 vs. 1, 3	0.081	83.7%
Frequent making of difficult decisions		46.6%	54.9%	49.8%	59.3%	<0.001 4 vs. 1, 2, 3	0.070	52.9%
Frequent management of personal knowledge gaps		38.3%	49.6%	40.0%	50.1%	<0.001 1 vs. 2, 4 3 vs. 2, 4	0.073	44.0%
Frequent assumption of responsibility for others		48.3%	53.0%	47.8%	61.8%	<0.001 4 vs. 1, 2, 3	0.082	52.8%
Frequent convincing/negotiating with others		58.4%	62.7%	58.7%	67.8%	<0.001 4 vs. 1, 3	0.063	61.9%
Frequent occupational communication with others		91.9%	95.5%	93.8%	95.0%	0.152		94.1%
**Work demands**								
High time and performance pressure		52.7%	85.4%	52.0%	81.3%	<0.001 1 vs. 2, 3, 4 3 vs. 2, 4	0.191	65.1%
Frequent detailed work instructions		27.5%	29.1%	18.4%	23.1%	<0.001 3 vs. 1, 2, 4	0.079	22.3%
Frequent repetition of the same work task in all details		43.3%	42.9%	40.0%	38.7%	0.725		40.4%
Frequently learning new tasks		43.6%	52.6%	45.4%	54.0%	<0.001 4 vs. 1, 3	0.064	48.7%
Frequency: improving procedures or trying something new (often)		25.8%	34.7%	33.8%	38.1%	<0.001 1 vs. 3, 4 2 vs. 3, 4	0.074	34.2%
Frequent disruptions or interruptions		52.7%	76.1%	52.4%	71.1%	<0.001 1 vs. 2, 3, 4 3 vs. 2, 4	0.135	61.0%
Frequent production targets or time constraints		29.9%	45.9%	26.4%	39.1%	<0.001 1 vs. 2, 4 3 vs. 2, 4	0.103	33.0%
Frequently required to do something not trained for		8.7%	19.8%	8.8%	17.7%	<0.001 1 vs. 2, 4 3 vs. 2, 4	0.107	12.8%
Frequent multitasking		64.8%	81.7%	71.2%	83.4%	<0.001 1 vs. 2, 3, 4 3 vs. 2, 4	0.109	75.4%
High frequency of working at maximum capacity		22.8%	57.1%	15.9%	45.5%	<0.001 1 vs. 2, 3, 4 2 vs. 3, 4 3 vs. 4	0.240	30.8%
Frequent need to work quickly		33.9%	56.0%	30.3%	49.9%	<0.001 1 vs. 2, 3, 4 3 vs. 2, 4	0.147	39.8%
Frequent problems detaching from work		37.2%	66.8%	30.3%	51.6%	<0.001 1 vs. 2, 4 2 vs. 3, 4 3 vs. 4	0.162	42.0%
Frequently emotional demanding situations		23.2%	48.1%	19.3%	37.8%	<0.001 1 vs. 2, 3, 4 2 vs. 3, 4 3 vs. 4	0.158	28.9%
Degree of autonomy at work	instructed work	10.4%	10.2%	8.4%	8.2%	0.197		8.8%
	mainly independent	76.6%	72.5%	80.3%	81.2%			79.1%
	both equally	12.9%	16.9%	11.2%	10.5%			11.9%
**Working conditions: physiological**								
Physiological strain		2.45 (1.92)	2.86 (2.18)	2.34 (1.84)	2.79 (2.05)	0.827		2.55 (1.97)
Frequent standing work		41.9%	48.1%	44.6%	47.9%	0.646		45.7%
At least one hour of continuous sitting during work (often)		66.8%	60.4%	62.8%	64.3%	0.299		63.5%
Frequent heavy lifting (men: 20 kg, women: 10 kg)		12.1%	22.0%	13.0%	20.0%	0.001 < 0.01 1 vs. 2, 4 3 vs. 2, 4	0.063	16.1%
Frequent exposure to smoke, dust or chemical fumes		11.4%	8.2%	7.5%	9.1%	0.519		8.6%
Frequent environmental stressors (cold, heat, moisture, wetness, drafts)		14.8%	19.0%	11.6%	14.6%	<0.05 3 vs. 2, 4	0.050	13.8%
Frequent work with oil, grease or grime		12.1%	12.3%	9.4%	10.5%	0.452		10.4%
Frequent demanding handwork		32.9%	35.4%	29.1%	33.9%	0.449		31.8%
Frequently working in uncomfortable postures (bending, squatting, kneeling, overhead)		9.4%	14.9%	10.7%	15.4%	<0.05 3 vs. 4	0.051	12.5%
Frequently working under poor lighting conditions		10.7%	14.9%	8.6%	12.7%	<0.001 1 vs. 2, 4 3 vs. 2, 4	0.077	10.9%
Frequent work under noise		21.8%	34.0%	22.7%	28.2%	<0.001 1 vs. 2, 4 3 vs. 2, 4	0.072	25.6%
Often working with microorganisms (e.g., pathogens)		11.1%	16.4%	13.7%	22.2%	<0.001 4 vs. 1, 3	0.075	16.3%
Work predominantly performed outdoors		6.0%	4.1%	5.9%	6.1%	0.215		5.8%
**Working conditions: psychological**								
Psychological strain		3.85 (2.52)	5.00 (2.40)	3.36 (2.29)	4.32 (2.40)	0.399		3.91 (2.43)
Often plan and organize one’s own work		70.4%	63.5%	75.0%	68.1%	<0.05 3 vs. 2, 4	0.061	70.9%
Frequent influence on workload allocation		33.1%	13.5%	33.9%	16.9%	<0.001 1 vs. 2, 4 3 vs. 2, 4	0.126	26.1%
Often able to decide when to take breaks		67.2%	53.8%	63.6%	54.8%	<0.001 1 vs. 2, 3, 4 3 vs. 2, 4	0.082	60.2%
Frequency of feeling that one’s work is important (often)		74.8%	67.5%	75.2%	76.2%	<0.01 2 vs. 3, 4	0.062	74.6%
Frequently not informed in time about major decisions, changes or future plans		25.4%	32.7%	19.1%	26.7%	<0.001 1 vs. 2, 3, 4 3 vs. 2, 4	0.090	23.9%
Frequently lacking information necessary to perform tasks properly		16.7%	25.8%	11.7%	20.7%	<0.001 1 vs. 2, 3, 4 3 vs. 2, 4	0.105	16.8%
Frequently feeling part of a community at the workplace		69.3%	68.1%	78.8%	72.2%	<0.01 3 vs. 1, 2, 4	0.062	74.3%
Frequently experience good cooperation with colleagues		75.4%	67.7%	83.6%	77.5%	<0.001 2 vs. 3, 4 3 vs. 1, 4	0.082	78.8%
Frequently receive help and support from colleagues when needed		68.7%	56.9%	77.2%	64.5%	<0.001 1 vs. 2, 3, 4 3 vs. 2, 4	0.104	69.7%
Frequently receive help and support from the direct supervisor when needed		47.5%	32.5%	54.2%	35.8%	<0.001 1 vs. 2, 3, 4 3 vs. 2, 4	0.121	45.0%
Frequently receive praise and recognition from the direct supervisor for doing a good job		25.9%	13.3%	26.9%	20.3%	<0.001 1 vs. 2, 3 2 vs. 3, 4 3 vs. 4	0.114	23.1%

Note. Means (SD) and percentage reported. Physiological and psychological strain were constructed as a summative index from the frequency data of the respective working conditions (maximum sum = 11). For working conditions, only the percentages of participants who reported “frequently” are presented.

Finally, when we consider the group without exhaustion but with overload, they face some cognitively and emotionally demanding job requirements, such as learning new tasks (54), making difficult decisions (59%), and taking responsibility for others (62%), most commonly among the groups.

The effect sizes range from very weak to small.

No statistically significant differences in perceived autonomy at work were observed between exhausted and non-exhausted, overloaded and non-overloaded employees (Table 2). Also, for on-call work and occasional remote work, as well as for psychological strain, no significant difference between the groups was observed (Table 2).

### 3.3. Individual Characteristics

There were more women in the exhausted groups (>60%, Table 1). A higher proportion of employees with exhaustion reported not working in their preferred occupation (44–47%, Table 1).

The four groups did not differ significantly in most individual characteristics (Table 3), i.e., work addiction-like behavior, personality traits such as openness, conscientiousness, extraversion, agreeableness, and neuroticism (Table 3), and internal control attribution. Procrastination behavior and willingness to take risks were reported similarly by all groups.

The emotionally exhausted groups and the overloaded but non-exhausted showed a slightly higher external control attribution than the group with neither. Nonetheless, the effect sizes for both are very small.

The short vocabulary test was similarly performed by all groups investigated.

The willingness to take risks varied most in the group that was exhausted but not overloaded. Similar proportions of this group reported decreasing risk-taking over time (35%) or increasing it (45%, Table 3).

**Table 3:** Personality dimensions and cognitive performance (vocabulary test) in employees with or without work overload, and with or without treatment due to emotional exhaustion. German representative working sample (*N* = 2289). One-way ANOVA with Bonferroni-corrected post hoc comparisons, Chi-square test.

		Employed with Emotional Exhaustion		Employed Without Emotional Exhaustion		ANOVA and χ^2^ *p* Overall, Bonferroni-Corrected Post Hoc Group Comparisons With Significant Differences		
Perceived Overload		Without Perceived Overload (1) *n* = 298	With Perceived Overload (2) *n* = 268	Without Perceived Overload (3) *n* = 1017	With Perceived Overload (4) *n* = 706		Partial η^2^ and Cramérs’ V	All (*N* = 2289)
Personality Constructs								
Locus of control	internal	4.0 (0.8)	3.8 (0.9)	4.0 (0.8)	3.9 (0.8)	0.635		3.9 (0.8)
	external	2.6 (1.0)	2.6 (1.0)	2.3 (0.9)	2.6 (1.0)	0.012 < 0.05 3 vs. 1, 2, 4	0.003	2.5 (0.9)
Occupational self-efficacy		4.2 (0.6)	3.9 (0.6)	4.2 (0.5)	4.0 (0.6)	0.147		4.1 (0.6)
Work addiction	excessively	3.4 (0.8)	3.7 (0.7)	3.4 (0.8)	3.8 (0.7)	0.108		3.6 (0.8)
	compulsively	2.6 (0.9)	2.9 (0.9)	2.6 (0.8)	2.8 (0.9)	0.757		2.7 (0.8)
	overall	3.0 (0.8)	3.3 (0.7)	3.0 (0.7)	3.3 (0.7)	0.484		3.1 (0.7)
Big Five	openness	3.6 (0.8)	3.6 (0.8)	3.5 (0.8)	3.5 (0.8)	0.644		3.5 (0.8)
	conscientiousness	4.2 (0.6)	4.2 (0.6)	4.1 (0.6)	4.1 (0.6)	0.814		4.1 (0.6)
	extraversion	3.6 (0.6)	3.5 (0.6)	3.5 (0.5)	3.5 (0.5)	0.541		3.5 (0.5)
	agreeableness	3.5 (0.5)	3.5 (0.5)	3.5 (0.5)	3.5 (0.5)	0.303		3.5 (0.5)
	neuroticism	3.1 (0.5)	3.1 (0.6)	3.0 (0.6)	3.1 (0.6)	0.793		3.1 (0.6)
Procrastination		2.6 (1.3)	2.7 (1.2)	2.7 (1.2)	2.8 (1.2)	0.561		2.7 (1.2)
Willingness to take risks (current)		3.1 (1.0)	2.8 (0.8)	3.0 (0.8)	2.9 (0.9)	0.227		2.9 (0.9)
Willingness to take risks (at the end of school in comparison to current)	roughly equally	19.2%	32.1%	30.4%	32.0%	0.023 < 0.05 1 vs. 4	0.084	29.8%
	much more	45.4%	37.6%	36.9%	42.8%			39.9%
	much less	35.4%	30.3%	32.7%	25.2%			30.4%
Intellectual pragmatics: vocabulary test (animal-naming task)		28.1 (8.1)	27.5 (7.2)	28.9 (7.3)	28.7 (7.4)	0.741		28.6 (7.4)

Note. Means (SD) and percentage reported. If percentage values are missing overall, it is due to missing data. Each personality dimension (exception: vocabulary test) is rated from 1 = *strongly disagree* or *not willing to take risks* to 5 = *strongly agree* or *very willing to take risks*.

## 4. Discussion

This analysis of a nationally representative employee sample across occupational sectors investigated a broad range of personal and work-related factors potentially associated with emotional exhaustion and work overload, and, consequently, may be relevant to work ability. Specifically, this analysis compared the four groups: “emotional exhaustion with perceived work-related overload”, “emotional exhaustion without overload”, “no emotional exhaustion but work-related overload”, and “neither of these” regarding specific working conditions and demands, as well as employee characteristics. This can provide a basis for further studies and may contribute to the development of possible preventive interventions targeting relevant risk and protective factors.

### 4.1. Work Characteristics and Individual Characteristics

First, taking into account the low correlation of the two phenomena, work overload and exhaustion should not be equated: they are distinct phenomena, and work overload may occur independently of exhaustion. This is consistent to a certain extent with previous research findings, as the two phenomena have rarely been considered separately—rather, they have typically been examined within the context of moderator and mediator models.

Second, the data partially support previous findings regarding emotional exhaustion, which report that emotional exhaustion is associated with performance, effective work behavior, and commitment. Those who are exhausted and overloaded are less likely to be in their preferred jobs, feel less that their work is important, feel less involved in shaping their work, and feel less socially connected. This is also consistent with the person-job fit model introduced at the beginning [2,15].

Third, the type of work demands—organizational or psychosocial—seems to be relevant: it is, for example, interesting that there were hardly any differences in remote work or on-call work rates among the four groups [37]. Seemingly, remote or on-call work does not appear to be a key indicator of overload or exhaustion. Also, the assumed impact of autonomy [10,38,39] on exhaustion and overload cannot be supported by the findings: No differences in perceived autonomy were observed between those who are exhausted and overloaded and others. This finding does not align with earlier studies, which discuss low autonomy as a major contributor to exhaustion and burnout [39]. A notable difference, however, is that demanding psychosocial work aspects showed a more systematic pattern of co-occurrence with exhaustion or overload, whereas physical work context conditions did not show a comparable pattern. Overloaded employees stated working in personal service occupations and higher professional/managerial positions more often and reported more psychosocial work demands at the same time, such as time and performance pressure, responsibilities, or the need for frequent problem-solving. In line with the literature, findings suggest that low perceived social support and low autonomy in work organization may be indicators of exhaustion and overload. Both the ability to ask for support and the ability to make use of one’s right to have a voice can be important resources for coping with the work demands in accordance with the JD-R model [40,41].

Fourth, exhaustion seems to specifically come along with the feeling of not being in the preferred occupation. Another specificity is that employees in treatment for exhaustion were more often women, and more often single, which is consistent with evidence on people with mental health issues [42,43]. Other reasons for mental health issues may include, for example, healthier lifestyles, better access to the healthcare system, or different contextual factors [44].

Finally, personality traits did not significantly differ between employees with and without exhaustion, overloaded or non-overloaded. There is thus no “exhaustion-prone” or “work overload-prone” personality pattern. These findings support the JD-R and person-job fit models, which both focus on the interplay of individual and work situation. Personality represents individual traits, which may fit in one work situation but may be perceived as an overload in another.

### 4.2. Non-Exhausted but Overloaded Employees

The non-exhausted but overloaded appear different from the exhausted and overloaded, and the others: the non-exhausted overloaded include the largest share of personal service employees and the smallest share in routine occupations. They are, due to their occupational profile, most often required to assume responsibility for others, make challenging decisions, and identify and resolve knowledge gaps. This finding may point to overload as such, representing a distinct profile that may be differentiated from exhaustion. Work overload thus appears to represent primarily an organizational factor which may, but need not, come along with emotional exhaustion, depending on the employee’s condition and perception [13,45].

Interventions aiming to prevent sick leave and work disability should therefore not only target employees with emotional exhaustion globally but may also need to address specific employee groups that appear to be prone to work-related overload in contexts of high psychosocial work demands.

### 4.3. Limitations

These analyses are based on pre-existing data from 2018. In this methodological context, it is common that the data are not perfectly tailored to the specific research questions, which may limit the interpretability of the results [46].

One of the grouping variables used was whether the employed individual was “receiving treatment for emotional exhaustion”, rather than the mere presence of emotional exhaustion symptoms. This is a more conservative definition of clinically relevant exhaustion problems, in contrast to a potentially inflation-like understanding of emotional exhaustion of any (even normal) type. Treatment for emotional exhaustion represents a more objective and robust indicator and therefore enhances the validity of exhaustion in the sense of “clinically relevant exhaustion”. Future research should consider structured diagnostic interviews for the assessment of mental health issues.

It can be discussed if the “overload” variable is appropriate, as it does not represent a substitute for work ability but rather an indicative factor. Furthermore, response options were dichotomized broadly into overloaded and non-overloaded categories. Future research could additionally consider the opposite phenomenon of boredom to provide an even more differentiated perspective.

The proportion of employees holding a university entrance qualification is already quite high (60.5%), which is due to the fact that solely employed people were investigated, neglecting many others (unemployed, people on disability pension, older age groups).

This present broad analysis did not cluster employees differentiated by occupation, sector, or workplace context. Multilevel modeling approaches could be applied in future analyses in order to explore the impact of specific conditions.

Interestingly, psychological strain resulting from work demands appears to be relatively similar across all groups, which could potentially reflect a Type I error. The reasons underlying this finding warrant further investigation. There are marginally to small effect sizes, suggesting that differences in such characteristics may have rather small practical relevance. In large samples, even small effects tend to become statistically significant. However, the pattern of characteristics and the differentiation of exhaustion and overload are of value.

Another important point is that the data is based on self-reports from employed individuals. Accordingly, no conclusions can be drawn about real work settings. Nevertheless, they offer the benefit of directly representing the individual’s perspective, which is known to impact work motivation and sick leave [47,48]. Behavioral observations or observer ratings from workmates or supervisors could help overcome this limitation.

A final limitation is that much of the data is nominal, the lowest measurement level, providing rather broad information on equality or inequality. This likewise constitutes the rationale for the selected analytical methods and the research question. Multivariate analyses may be useful to enable stronger inferences regarding potential causal relationships and to address the risk of Type I error bias, particularly through the use of multinomial logistic regression. Due to data quality limitations (cross-sectional) and the descriptive, exploratory nature of the present approach (comparison of four defined, distinct groups), we did not apply causality-implying methods.

The dataset’s greatest strength lies in its extensive coverage of both work-related and personal variables in a sample representative of the German population, a level of comprehensiveness rarely achievable with other data collection methods.

## 5. Conclusions

Exhaustion and work overload can occur independently in employees and should not be mixed up. Overload appears to be associated with psychosocial work demands, whereas exhaustion appears to be more related to specific individual aspects: female gender and perception of insufficient person-job fit.

The group characterized by overload but without exhaustion appears to be particularly burdened by overload-related factors, and most individuals in this group seem to come from the “personal services” sector. Accordingly, this topic warrants further research. For future research, we recommend focusing more on causal relationships and recruiting longitudinal data.

The differential patterns observed in employees with overload or exhaustion should be taken into account in individualized occupational psychosomatic consultation services [49].

## Data Availability

The data and material presented in this paper are available as a Scientific Use File (SUF) upon submission of a data request (at https://www.bibb.de/de/1386.php (accessed on 10 April 2026)) to the BIBB Research Data Center (BIBB FDZ). The data are provided by GESIS–Leibniz Institute for the Social Sciences, Cologne. https://doi.org/10.7803/501.18.2.1.10.

## Abbreviations

The following abbreviations are used in this manuscript:

BAuA	Federal Institute for Occupational Safety and Health: “Bundesanstalt für Arbeitsschutz und Arbeitsmedizin”
BIBB	Federal Institute for Vocational Education and Training: “Bundesinstitut für Berufsbildung”
JD-R model	Job Demands-Resources model
nE	no/without emotional exhaustion
nO	no/without perceived overload
wE	with emotional exhaustion
wO	with perceived overload
